# Supplementary figures and images for: Metagenomic survey of methanesulfonic acid (MSA) catabolic genes in an Atlantic Ocean surface water sample and in a partial enrichment
Source: PeerJ. 2016 Oct 6;4:e2498. doi: 10.7717/peerj.2498 (PMC5068391; doi:10.7717/peerj.2498)

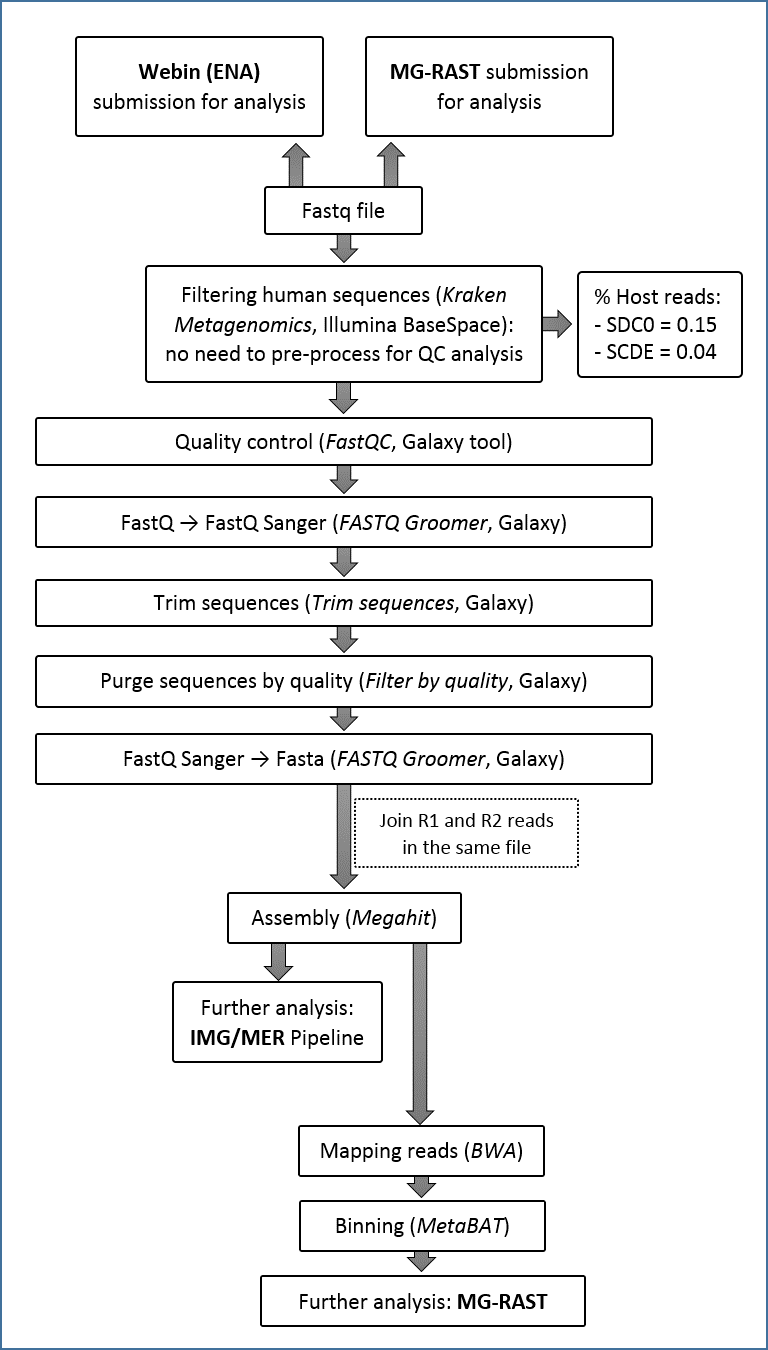

Supplement: Figure S1 [file peerj-04-2498-s001.png]

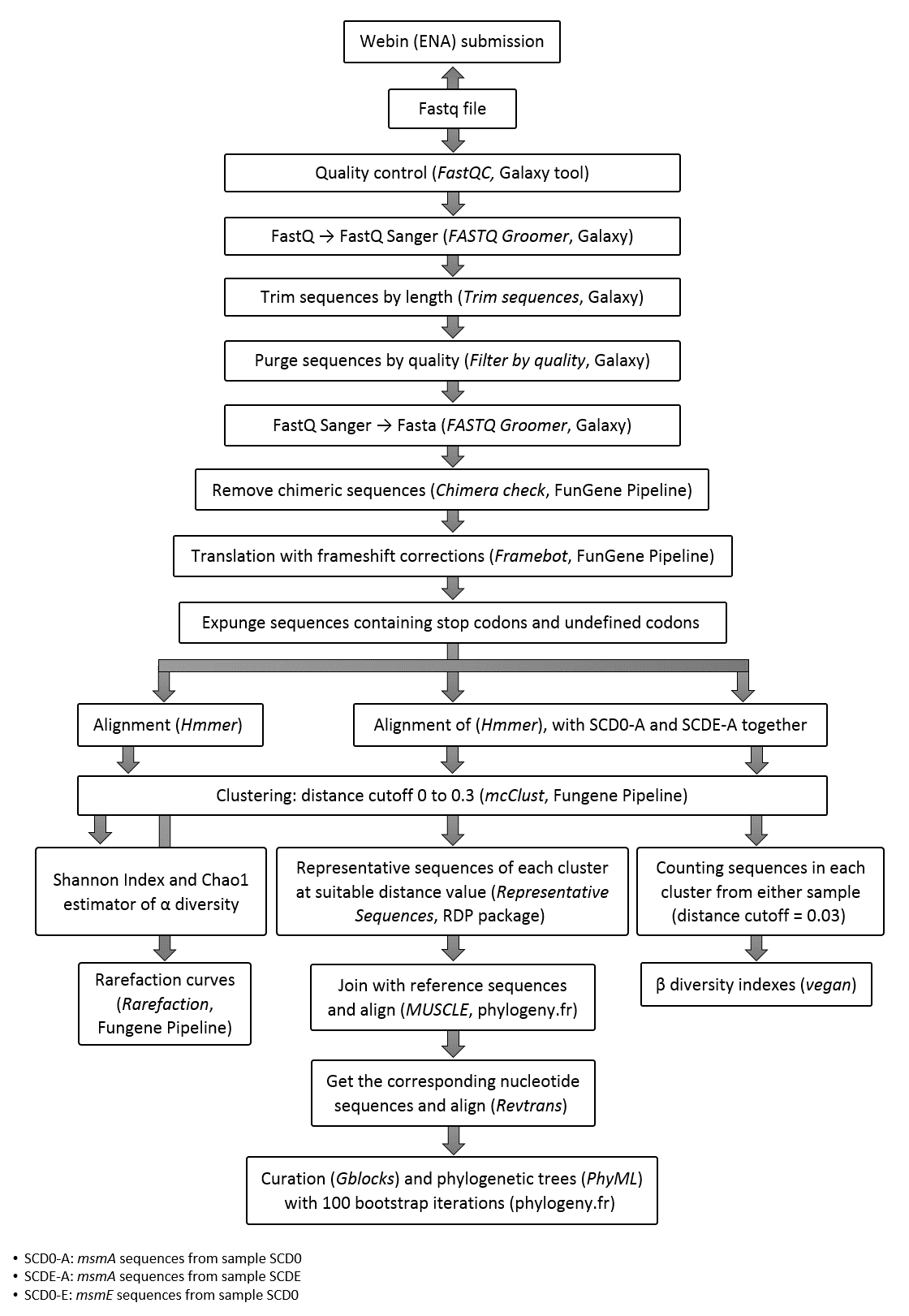

Supplement: Figure S2 [file peerj-04-2498-s002.png]

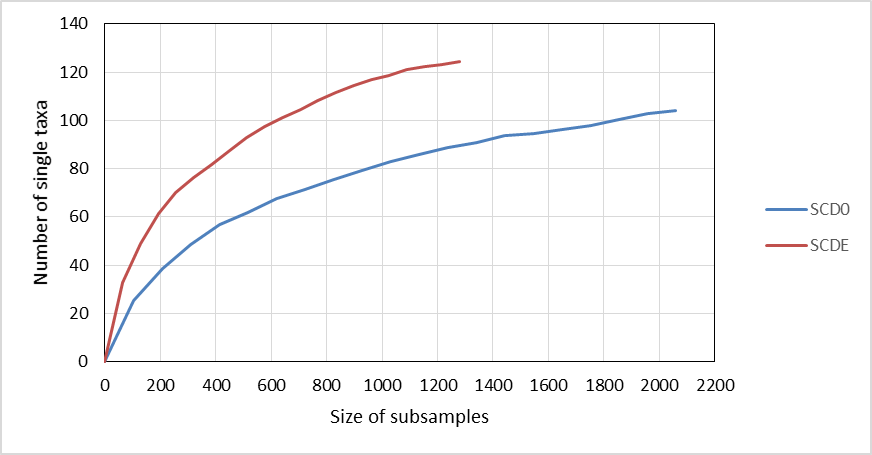

Supplement: Figure S3 [file peerj-04-2498-s003.png]
