# Supplementary material for: Metagenomic survey of methanesulfonic acid (MSA) catabolic genes in an Atlantic Ocean surface water sample and in a partial enrichment
Source: PeerJ. 2016 Oct 6;4:e2498. doi: 10.7717/peerj.2498 (PMC5068391; doi:10.7717/peerj.2498)
Supplement: Table S1 [file peerj-04-2498-s004.docx]

Table S1. General statistics of whole metagenome sequencing reads quality control performed by EBI Metagenomics.

| Sample | Raw data  (nº of sequences) | After quality processing | | | |
| --- | --- | --- | --- | --- | --- |
|  |  | Number of sequences | Mean sequence length (bp) ± SD | Average GC% | Low-quality sequences discarded |
| SCD0 | 14,246,188 | 10,620,194 | 103.1 ± 11.7 | 43.30 | 3,625,994 (25%) |
| SCDE | 15,660,864 | 10,980,517 | 102.6 ± 10.6 | 53.29 | 4,680,347 (30%) |
