# Supplementary material for: Metagenomic survey of methanesulfonic acid (MSA) catabolic genes in an Atlantic Ocean surface water sample and in a partial enrichment
Source: PeerJ. 2016 Oct 6;4:e2498. doi: 10.7717/peerj.2498 (PMC5068391; doi:10.7717/peerj.2498)
Supplement: Table S2 [file peerj-04-2498-s005.docx]

Table S2. General statistics of whole metagenome sequencing reads quality control and assembly for IMG/MER submission.

| Sample | | Raw data | | After quality processing | | | After assembly | |
| --- | --- | --- | --- | --- | --- | --- | --- | --- |
|  |  | Number of reads | Mean reads length (bp) ± SD | Number of sequences | Mean sequence length (bp) | Low-quality sequences discarded | Number of sequences | Mean sequence length (bp) ± SD |
| SCD0 | R1 | 7,123,094 | 99.8 ± 5.6 | 6,314,501 | 85.2 ± 5.3 | 808,593 (11%) | 73,571 | 374 ± 303 |
|  | R2 | 7,123,094 | 99.8 ± 5.6 | 5,576,029 | 85.2 ± 5.7 | 1,547,065 (21%) |  |  |
| SCDE | R1 | 7,830,432 | 99.8 ± 5.5 | 6,581,133 | 81.2 ± 5.5 | 1,249,299 (15%) | 86,670 | 489 ± 703 |
|  | R2 | 7,830,432 | 99.8 ± 5.4 | 5,570,859 | 83.2 ± 5.3 | 2,259,573 (28%) |  |  |
