# Supplementary material for: Metagenomic survey of methanesulfonic acid (MSA) catabolic genes in an Atlantic Ocean surface water sample and in a partial enrichment
Source: PeerJ. 2016 Oct 6;4:e2498. doi: 10.7717/peerj.2498 (PMC5068391; doi:10.7717/peerj.2498)
Supplement: Table S3 [file peerj-04-2498-s006.docx]

Table S3. Primers used in the amplification of *msmA* and *msmE* gene sequences from SCD0 and SCDE samples.

|  | **Primer name** | **Sequence (5’→3’)** |
| --- | --- | --- |
| **Primers aimed at *msmA* sequence** | SarA124fwd | AAAAACGTCTGGGTTCCAGTTTGTC |
|  | SarA1053rev | CAAATCTTCATGCAGATTCCTTCC |
|  | SarA139fwd | CCAGTTTGTCACGAATCCG |
|  | SarA488rev | ACAAATCCACCATATTTCACTTCAC |
| **Primers aimed at *msmE* sequence** | SarE133fwd | TTCGGGAAACCTGGAGAACCTGTAAATTTGG |
|  | SarE1125rev | GCCAAGTGGACTCTTTAAGCCACGATCTGC |
|  | SarE322fwd | CATATTGGATACATGGGTGACATGCCC |
|  | SarE704rev | GCTGCTCTTCTGGCAATACCTAG |
