# Supplementary material for: Metagenomic survey of methanesulfonic acid (MSA) catabolic genes in an Atlantic Ocean surface water sample and in a partial enrichment
Source: PeerJ. 2016 Oct 6;4:e2498. doi: 10.7717/peerj.2498 (PMC5068391; doi:10.7717/peerj.2498)
Supplement: Table S4 [file peerj-04-2498-s007.docx]

Table S4. Successful PCR conditions for the amplification of *msmA* and *msmE* gene sequences from SCD0 and SCDE samples.

|  | **Nested PCR for amplification of *msmA* sequences** | | | **Nested PCR for amplification of *msmE* sequences** | |
| --- | --- | --- | --- | --- | --- |
|  | **Primers SarA124fwd/SarA1053rev**  **(First PCR)** | | **Primers SarA139fwd/SarA488rev (Second PCR)** | **Primers SarE133fwd/SarE1125rev**  **(First PCR)** | **Primers SarE322fwd/SarE704rev**  **(Second PCR)** |
| **Metagenome^a^** | SCD0 | SCDE | SCD0 and SCDE | SCD0 and SCDE | SCD0 and SCDE |
| **PCR program** | 95^o^C 2 min + 35 x (94^o^C 1 min + 56.2^o^C 1 min + 72^o^C 1min) + 72^o^C 5 min | 95^o^C 2 min + 35 x (94^o^C 1 min + 52.1^o^C 1 min + 72^o^C 1min) + 72^o^C 5 min | 95^o^C 2 min + 30 x (94^o^C 1 min + 51^o^C 30 seg + 72^o^C 1min) + 72^o^C 5 min | 95^o^C 2 min + 35 x (94^o^C 1 min + 58.2^o^C 1 min + 72^o^C 1min) + 72^o^C 5 min | 95^o^C 2 min + 30 x (94^o^C 1 min + 55^o^C 30 seg + 72^o^C 1min) + 72^o^C 5 min |
| **Concentrations^b^** | 0.8 µM of forward and reverse primers, 1.25 U of GoTaq® G2 Flexi DNA polymerase (Promega). | 0.8 µM of forward and reverse primers, 1.25 U of GoTaq® G2 Flexi DNA polymerase (Promega), with 0.125 M betaine and 2.5% DMSO | 0.8 µM of forward and reverse primers, 1.25 U of GoTaq® G2 Flexi DNA polymerase (Promega). | 0.8 µM of forward and reverse primers, 1.25 U of GoTaq® G2 Flexi DNA polymerase (Promega). | 0.8 µM of forward and reverse primers, 1.25 U of GoTaq® G2 Flexi DNA polymerase (Promega). |

(^a^) The DNA was first amplified using REPLI-g® MiniKit (QIAGEN)

(^b^) In all cases, PCR was performed in a 25 μL volume using the manufacturer’s buffer associated with the Taq polymerase employed, 1.5 mM MgCl_2_ and 200 µM of each dNTP.
