# Supplementary material for: Metagenomic survey of methanesulfonic acid (MSA) catabolic genes in an Atlantic Ocean surface water sample and in a partial enrichment
Source: PeerJ. 2016 Oct 6;4:e2498. doi: 10.7717/peerj.2498 (PMC5068391; doi:10.7717/peerj.2498)
Supplement: Table S5 [file peerj-04-2498-s008.docx]

Table S5. General statistics of amplicon survey quality control.

|  | Raw data | Nucleotide sequences after quality processing | | | Number of sequences after frameshift and stop-codon correction | Low-quality sequences discarded |
| --- | --- | --- | --- | --- | --- | --- |
|  |  | Number of sequences | Mean sequence length ± SD (bp) | Percentage of GC (%) |  |  |
| SCD0-A | 230,221 | 39,297 | 343.7 ± 14.7 | 38.93 | 31,696 | 198,525 (86.2%) |
| SCDE-A | 168,403 | 55,519 | 346.9 ± 10.2 | 38.57 | 42,508 | 125,895 (74.8%) |
| SCD0-E | 115,970 | 12,653 | 377.9 ± 12.0 | 41.24 | 8,273 | 107,697 (92.9%) |
